# Power generating reflective-type liquid crystal displays using a reflective polariser and a polymer solar cell

**DOI:** 10.1038/srep11558

**Published:** 2015-06-23

**Authors:** Yoon Ho Huh, Byoungchoo Park

**Affiliations:** 1 Department of Electrophysics, Kwangwoon Univ., Wolgye-Dong, Nowon-gu, Seoul 139-701, Korea

## Abstract

We herein report the results of a study of a power generating reflective-type liquid crystal display (LCD), composed of a 90° twisted nematic (TN) LC cell attached to the top of a light-absorbing polymer solar cell (PSC), i.e., a Solar-LCD. The PSC consisted of a polymer bulk-heterojunction photovoltaic (PV) layer of poly[[9-(1-octylnonyl)-9H-carbazole-2,7-diyl]-2,5-thiophenediyl-2,1,3-benzothiadiazole-4,7-diyl-2,5-thiophenediyl] and [6,6]-phenyl C_71_ butyric acid methyl ester (PCDTBT:PCBM_70_), and showed a high power conversion efficiency of about 5%. In order to improve the visibility of the Solar-LCD, between the TN-LC and the PV cells we inserted a reflective polariser of a giant birefringent optical (GBO) film. The reflectivity from the Solar-LCD was observed to be considerably increased by more than 13–15% under illumination by visible light. The Solar-LCD also exhibited a significantly improved contrast ratio of more than 17–19. We believe there is a clear case for using such Solar-LCDs in new power-generating reflective-type displays; taken as a whole these results also demonstrate the possibility of their application in a number of energy-harvesting opto-electrical display devices.

Liquid crystal displays (LCDs) are now one of the most prevalent electronic information display devices in everyday use^1^. To improve their image quality and contrast ratio, LCDs have been developed and designed to absorb incident light selectively; for example, polarisers in LCDs must reliably absorb the unwanted polarisation component of backlight or ambient light^1^,^2^. However, despite a number of major developments aimed at reducing the significant wastage of light energy in LCDs, much of this energy is still wasted^3^. For instance, around 75% of the backlight energy is lost to the polariser in conventional LCDs, and the light absorbed is then wasted as heat^3^. Recently, a number of approaches have been used to try to scavenge energy from the LCD using polymer solar cells (PSCs)^4^,^5^,^6^ or a luminescent solar concentrator (LSC) system^7^. Based on the photovoltaic (PV) performance of PSCs as highlighted over the past decade, together with the development of new methods of fabrication and characterisation to achieve low-cost solar energy harvesting^8^,^9^,^10^,^11^, polarising PV effects in oriented PSCs^4^,^5^,^6^ and related structures^12^,^13^ have been investigated for power-generating polariser applications in LCDs (Solar-LCDs). Such in-plane anisotropic PSC devices may be applied to energy-harvesting applications; however, the device performance of these devices is still quite poor and image quality is very low (as low as ~1.7 in terms of contrast ratio). PV performance is also not good enough (about ca. 2.1% in terms of power conversion efficiency (PCE)); such device performance therefore needs to be improved further for most practical applications^4^,^5^,^6^. In order to use Solar-LCDs as practical energy-harvesting displays, a contrast ratio of at least 12–18 is needed, in order to achieve visibilities comparable with those of conventional reflective-type LCD devices^14^. Therefore, despite recent developments, new methods for recycling or harvesting the absorbed energy to generate electric power without losing contrast ratio or image quality are clearly required, potentially resulting in revolutionary, energy-saving LC devices. Such functionality would be especially promising considering the widespread use of devices such as mobile information displays (e.g., mobile phones), which are in standby mode for about 95% of the time.

We herein describe the use of an isotropic organic semiconductor as a power-generating integral part of a specially designed reflective-type dual-functional LC device operating as an attractive reflective-type display and simultaneously converting the absorbed light into electricity. Such reflective-type devices are superior to transmissive-type devices for outdoor use and can operate under direct sunlight for more energy absorption and energy conversion using the scheme discussed here. The dual functionality of our conceptual device is achieved by introducing a reflective polarising component combined with a PSC to make polarising PV panels in light-energy-harvesting LCDs. The polarisation control of the reflective polarising components and their unique applications in photonics were highlighted following the development of a giant birefringent optical (GBO) film^15^. The polarising optical effects of GBO films in LCDs^16^ and organic light-emitting diodes (OLEDs)^17^,^18^,^19^ have also been exploited to enhance the performance of display devices.

In proof of the concept involved, we demonstrate the principle of our reflective-type Solar-LC display capable of generating power by recycling the wasted light energy in the displays using a combination of a PSC and a reflective GBO polarising film, which transmits light selectively and transports it to the back of the device, where the isotropic PSC converts it into electricity. This approach exploits the principles of a reflective-type solar-LCD in harvesting energy from ambient light or sunlight while hiding the PSCs at the back of the device, leaving the entire front surface available for the display without sacrificing the contrast ratio or degrading image quality. To the best of our knowledge this is the first time a GBO reflecting polariser has been used to construct a reflective-type Solar-LCD. Furthermore, we adopt PSC structures for our dual-function devices, and the approach thus also takes advantage of the low cost of the PSC, as well as its easy fabrication and compatibility with flexible substrates over a large surface area^9^,^10^,^11^. This study also points to alternative applications of PSCs, complementing the concerted efforts currently being made to improve their PCEs^20^,^21^,^22^,^23^,^24^,^25^,^26^,^27^,^28^,^29^,^30^,^31^,^32^ and practical fabrication^33^,^34^,^35^,^36^,^37^. In addition, unlike previous linearly rubbed PSCs^4^,^5^,^6^, the isotropic PV layer used in this Solar-LCD does not suffer from any physical damage caused by mechanical rubbing leading to possible deterioration in device performance. This rub-free process is important in improving the PV performance of Solar-LCDs, because almost none of the highly efficient PSCs developed recently can endure severe rubbing due to the soft surface of the PV layers. The results of the present study could therefore represent a crucial step forwards in practical energy harvesting in bendable, flexible, or wearable LC display devices.

## Experimental

The LC used in this study was a nematic LC mixture (ZLI-2293, Merck) with a nematic phase below the clearing point of 85 °C. The extraordinary (*n*_e_) and ordinary (*n*_o_) refractive indices of ZLI-2293 are 1.6313 and 1.4990 (λ = 589.3 nm) at room temperature, respectively. An empty LC cell was made up by sandwiching together two conductive indium-tin-oxide (ITO) coated glass substrates, with a cell gap maintained at 4.2 μm using glass spacers. The surfaces of the ITO layers were coated with rubbed polyimide alignment layers; as a result the LC molecules became oriented parallel to the direction of rubbing. The rubbing directions of the two substrates were perpendicular, such that a 90° twist of the LC director from one substrate to the other was promoted inside the cell. By capillary-filling the empty cell with ZLI-2293, we obtained a reflective twisted nematic (TN) LC cell. The 90° twist results in a 90° rotation of the polarisation of the light after it passes through the LC cell, as in waveguide mode^38^.

For the PSCs, poly[[9-(1-octylnonyl)-9H-carbazole-2,7-diyl]-2,5-thiophenediyl-2,1,3-benzothiadiazole-4,7-diyl-2,5-thiophenediyl] (PCDTBT, 1-material Chemscitech Inc.) and [6,6]-phenyl C_71_ butyric acid methyl ester (PCBM_70_, Nanostructured Carbon Inc.) were used as received. The PSCs were fabricated as follows. First, a clean ITO layer (80 nm, 30 ohm/square) on glass was used as the anode. Routine cleaning of the ITO substrate in an ultrasonic bath of acetone for 10 min was followed by rinsing with deionised water, drying with nitrogen, and ultra-violet ozone cleaning. After that, a blended solution of PCDTBT (0.456 wt%) and PCBM_70_ (1.824 wt%)^39^ was spin-coated on the ITO precoated with a hole-collecting poly(3,4-ethylenedioxythiophene): poly(styrene sulfonate) (PEDOT:PSS, CLEVIOS^TM^ 4083, H. C. Starck Inc., 40 nm) buffer layer. The PCDTBT:PCBM_70_ PV layer was about 85 nm thick. After spin coating, the PV layer was annealed at a temperature of 65 °C for 1 hour. Finally, 1 nm Al:Li alloy (Li: 0.1 wt%) / Al cathode (50 nm) layers were then formed on the PCDTBT:PCBM_70_ PV layer by thermal deposition (0.5 nm/s) at a base pressure of less than 2.7 × 10^−4^ Pa. The structure of the PSC was therefore [ITO/PEDOT:PSS/ PCDTBT:PCBM_70_/Al:Li/Al]. All the fabricated PV cells were then tested under ambient conditions without encapsulation. The PV performance was measured using a source meter (2400, Keithley) and calibrated using a reference cell (BS-520, Bunkoh-keiki) under an illumination of 100 mW/cm^2^ produced by an AM 1.5G light source (96000 Solar Simulator, Newport). The incident photon-to-current collection efficiency (IPCE) spectra were obtained using a measurement system (Oriel^®^ IQE-200™, Newport).

To fabricate the reflective Solar-LCD devices, the TN-LC cell, which was placed between a front sheet dichroic polariser at the input end and the rear GBO reflecting polariser at the reflecting end, was attached to the PCDTBT:PCBM_70_ PSC. The polarisation (passing) axis of the front sheet dichroic polariser was parallel to the LC director of the TN-LC cell at the input end, and the passing axis of the rear GBO polariser was set parallel to the passing axis of the front sheet polariser, in order to achieve normal white (NW) mode^38^ operation. The illuminating light was normally incident to the Solar-LCD. The optical characteristics of each component of the Solar-LCD were investigated using polarised microscopy via a UV-visible spectroscopy system (8453, Agilent).

## Results and Discussion

**Figure 1:**
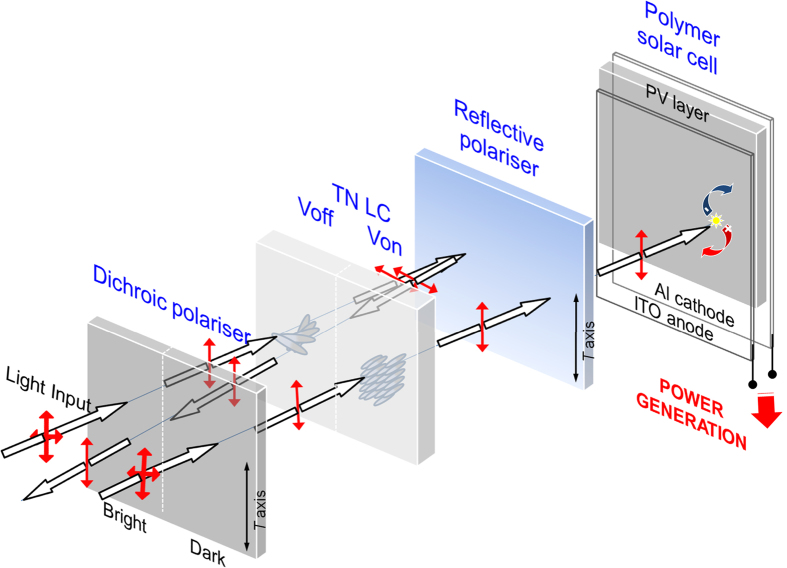
Schematic illustration of the bright (left) and dark (right) pixels of the reflective-type Solar-LCD, consisting of a front linear dichroic sheet polariser, TN-LC cells, a rear reflective polariser, and a polymer solar cell in the normally white mode. The white arrows indicate the propagation of incident lights and the red arrows (↕) represent the polarisation directions of the propagating lights. The *T* axis shown indicates the passing axis of the polarisers.

Figure 1 shows an exploded schematic illustration of the reflective-type Solar-LCD investigated in this study. The Solar-LCD includes, in order, a bottom light-absorbing isotropic PSC, a rear GBO reflective polariser, top TN-LC cells, and a front or light-exiting dichroic linear sheet polariser. Here, the top TN-LC cells, in which a 90° twisted orientation of the LC molecules is used with cell gap *d*, birefringence Δ*n*, and electrodes placed between the substrates, may be a light valve or an LCD having a matrix array of pixels and coloured (*i.e.*, RGB) subpixels. Two modes of operation of the Solar-LCD device may be possible, depending on the orientation of the passing axis of the GBO polariser. When the passing axis of the rear GBO polariser is parallel to that of the front polariser (parallel-polariser condition), the device can operate in the NW mode. When no voltage is applied (the “OFF” state, left pixel in TN-LC), after passing through the front dichroic polariser the polarised light enters the TN-LC cell, where the twisted orientation of the LC molecules causes the light to change its polarisation by 90° under the Mauguin condition^40^ with a retardation value >> 0.5 λ for an incident light of wavelength λ, in waveguide fashion, making it perpendicular to the passing axis of the rear GBO reflecting polariser. The light that emerges from the TN-LC cell is thus blocked and reflected at the GBO polariser, because here its polarisation is perpendicular to the passing axis of the GBO polariser in this bright state. In contrast, when a voltage is applied to the TN-LC cell (the “ON” state, right pixel in the TN-LC), the LC molecules begin to align along the electric field due to the positive dielectric anisotropy of the LC. At a sufficiently high field, the 90° twist is removed, and the polarisation of the light passing through the TN-LC cell does not change. Thus, the light that passes through the TN-LC cell passes through the second reflective polariser, implying that the rectilinearly propagating light is absorbed by the bottom PSC and contributes to the power generation such that the TN-LC cell is observed to be in the black state. The reflected output of the system therefore ranges from the bright reflection state to the dark extinction state, depending on the voltage applied to the TN-LC cell. The grey scale of the reflected output of the system is achieved by applying intermediate voltages between zero and the value at which light is completely transmitted. We also note that if the passing axis of the rear reflective polariser is perpendicular to that of the front polariser (cross-polariser condition), light is then absorbed (dark) at the field-off state and reflected (bright) at the field-on state (the normal black (NB) mode). In addition to its display ability, as mentioned above the light-absorbing bottom (unrubbed) PSC in the Solar-LCD can generate electricity using the selectively absorbed light. This allows our Solar-LCD to be used as a new power-generating reflective-type LC display, in which the entire surface is available for use in the display with a high contrast ratio and a high PV performance, unlike conventional reflective LC displays or conventional rubbed Solar-LCDs.

**Figure 2:**
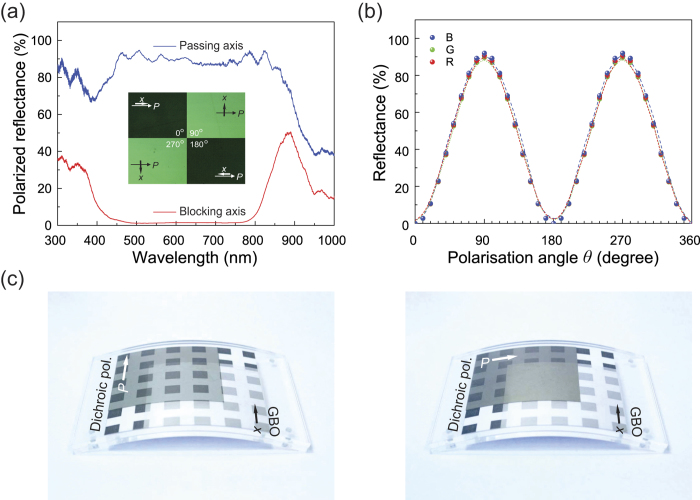
(**a**) Polarised reflectance spectra of the GBO reflecting polariser for incident light polarised linearly along the passing and reflecting axes. The inset shows the reflected–light photomicrographs at four angles of rotation of the GBO polariser under an optical polarising microscope. The arrows indicate the passing axis (*x*) of the GBO polariser and the orientation (*P*) of the polariser of the microscope. (**b**) Polarised reflectance of the GBO polariser as a function of the rotating angle θ between the polarisation direction of incident light and the passing axis of the GBO polariser for blue (470 nm), green (550 nm) and red (630 nm) lights. (**c**) Photographs of a pair of flexible polarisers that are partially overlapping, showing an underlying GBO reflecting polariser and an overlying dichroic sheet polariser for parallel (left) and crossed (right) polarisation states. The arrows show the passing axes of the polarisers.

We used a GBO polarising film as the reflective polariser in the Solar-LCDs. The polarised reflectance spectra from the GBO reflecting polariser were then observed for the two incident light beams polarised linearly along the passing and reflecting axes, both of which are shown in Fig. 2(a). From the figure, it is clear that the nature of the reflection bands depends strongly on the polarisation of the incident light; when measured in the direction of the reflecting axis, the reflection spectrum shows a strong and broad reflection band, while in the direction of the passing axis, there is virtually no reflection band in the wide visible wavelength range (400 ~ 800 nm) that includes blue (B), green (G), and red (R) lights. The average extinction ratio of the GBO reflective polariser used was estimated to be about 60:1 for wavelengths between 450 and 750 nm. The polarised reflection from the GBO polariser can be seen in the reflective microphotograph of the polariser obtained under polarised incident light for four angles of rotation of the GBO polariser (Inset in Fig. 2(a)). This figure confirms that the GBO film used causes a clear polarised reflection of light. The two darker views of the reflective microphotographs enable us to obtain the orientations of the optic axes for the GBO reflecting polariser. Figure 2(b) shows a graph of the polarised reflectance of the GBO reflecting polariser as a function of the azimuth rotation angle *θ* between the passing axis of the GBO polariser and the polarisation of the incident light for B (470 nm), G (550 nm), and R (630 nm) lights at normal incidence. As shown in the figure, the reflectance *R* is clearly governed by the relationship *R* = *A* cos^2^(90°−*θ*), where *A* is the polarised reflectance for incident light polarised linearly along the reflecting axes.

**Figure 3:**
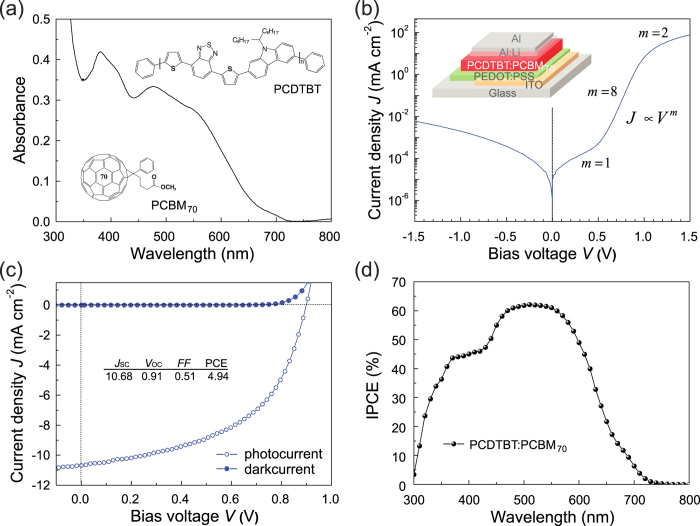
(**a**) The optical absorption spectra of the PCDTBT:PCBM_70_ BHJ PV layer (~85 nm). The insets show the molecular structures of PCDTBT and PCBM_70_. (**b**) Semilogarithmic plot of the dark current density-voltage (*J-V*) curve of the PCDTBT:PCBM_70_ PSC. The inset shows the schematic structure of the PCDTBT:PCBM_70_ BHJ PSC. (**c**) *J-V* curves of the PCDTBT:PCBM_70_ PSC under illumination (open symbols), together with the curves under dark conditions (closed symbols). The inset summarises the performance characteristics of the PSC. (**d**) IPCE spectra of the PCDTBT:PCBM_70_ PSC.

We will now discuss the optical performance of the GBO polariser when combined with a dichroic linear polariser. Figure 2(c) shows photographs of the overlapping dichroic sheet polariser on the underlying GBO polariser (8.5 × 8.5 cm^2^) under incident light originating from the ambient environment for two orientations of the polarisers; in the figures their passing axes are parallel (left-hand panel) and perpendicular (right-hand panel) to each other. When the passing axes of the polarisers are parallel (left-hand panel), in the region where the GBO and linear dichroic polarisers overlap the polarised light passes through the rear GBO polariser after passing through the front dichroic polariser; the black squares on the underlying paper may therefore be seen fairly clearly, representing the “dark” state (as in the light-absorbing black squares). In contrast, when the passing axes of the polarisers are perpendicular (right-hand panel of Fig. 3(c)), the light that passes through the front polariser is reflected at the rear GBO polariser, because its polarisation is parallel to the reflecting axis of the GBO polariser. The black squares on the paper behind cannot therefore be seen, indicating a “bright” state. Therefore, by adjusting the polarisation state of the propagating light, it is possible to control the reflected output of the system from the dark extinction state to the bright reflection state. To adjust the polarisation of the light, we used a simple voltage-controllable TN-LC cell, and the black squares on the paper were replaced by the light-absorbing PSC, as discussed below.

In the light-absorbing PSC, the polymer bulk-heterojunction (BHJ) PV layer was composed of a blend of PCDTBT and PCBM_70_, as is appropriate for low-band gap PSCs^21^,^39^. Figure 3(a) shows the chemical structure and optical absorption spectra of the PCDTBT:PCBM_70_ PV layer after annealing at 65 °C for 1 hour. The PCDTBT:PCBM_70_ PV layer showed strong absorption bands in the visible range from 350 to 650 nm, extending to an absorption onset at 720 nm, and containing two distinct but broad absorption bands centred at *ca.* 380–420 nm and *ca.* 475–570 nm. The two broad absorption bands with peaks at 398 and 576 nm were caused by the PCDTBT, and the absorption near 450 nm was caused by the PCBM_70_.

The structure of the PSC, as shown in the inset of Fig. 3(b), consists of a transparent ITO anode with a hole-collecting PEDOT:PSS buffer layer, a PCDTBT:PCBM_70_ PV layer, and an Al:Li/Al cathode. We then investigated the current density-voltage (*J–V*) characteristics of the fabricated PCDTBT:PCBM_70_ PSCs in the dark. Figure 3(b) shows the dark *J–V* characteristics of the fabricated PSC, demonstrating its good diodic behaviour with high rectification ratios of 10^4^ at 1.5 V. The PV characteristics of the PSCs were also investigated, as shown in Fig. 3(c). Under 100 mW/cm^2^, AM 1.5G simulated solar illumination, the PSC showed a good PV performance with an open-circuit voltage (*V*_*OC*_) of 0.91 V, a short-circuit current density (*J*_*SC*_) of 10.68 mA/cm^2^, and a fill factor (*FF*) of 0.51. These results correspond to a PCE of about 4.94%, which is comparable to values (*ca*. 4.9%) reported by others^39^. We further evaluated the PV performance of the PSCs by measuring the IPCE spectra. The observed IPCE spectra of the PSC devices are shown in Fig. 3(d). It is noteworthy that the absorption spectrum of the PCDTBT:PCBM_70_ PV layer (Fig. 3(a)) is responsible for the IPCE spectral shapes under illumination. The maximum IPCE was 62.1% at 510 nm for the PSC. These results confirm that the generation of photo-carriers is influenced by strong optical absorption in the PV layer, generating electron-hole pairs from the absorbed light and implying an efficient polymer PV cell. In addition, we also note that the PCDTBT:PCBM_70_ PV layer could not withstand rubbing, in other words the soft surface of the PV layer led to its serious damage or even its removal, clearly indicating that the previous Solar-LCD structure could not be achieved using a rubbed PCDTBT:PCBM_70_ PV layer.

**Figure 4:**
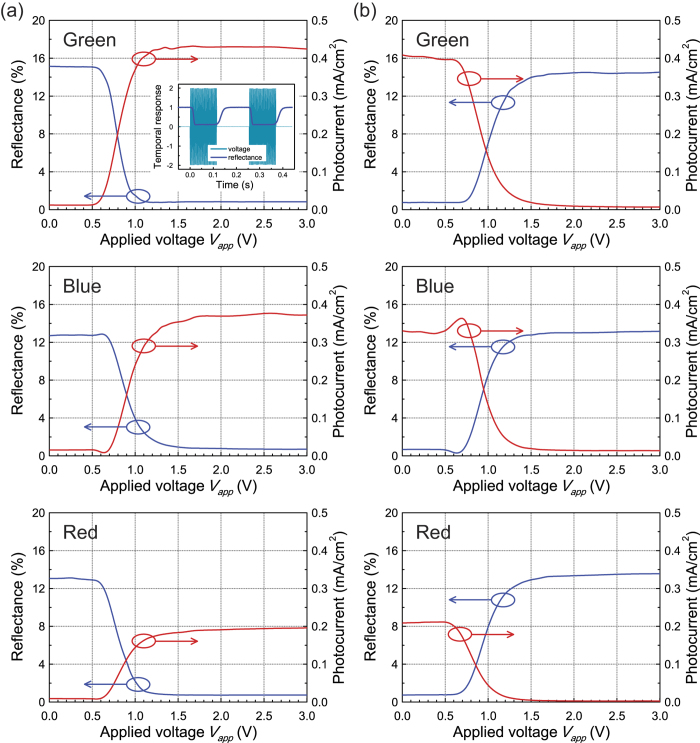
Voltage-dependent reflectance (blue curves) and short-circuit photocurrent density (red curves) characteristics of the reflective-type Solar-LCD in the normal white (a) and black (b) modes under green (upper), blue (middle), and red (lower) illumination. The inset in (**a**) shows the temporal responses of reflectance from the Solar-LCD with a 2.0 V square-wave voltage applied.

Next, in order to investigate the dual functionality of the reflective-type Solar-LCD, consisting of the GBO reflecting polariser between the TN-LC cell and the isotropic PCDTBT:PCBM_70_ PSC, we measured its electro-optical characteristics (Fig. 4). Figure 4(a) shows the reflectance characteristics of the reflected output from the device as a function of the voltage applied (*V*_*app*_) to the TN-LC cell in the NW mode under B(442 nm), G(532 nm), and R(633 nm) light. Here, the absolute reflectance is denoted by *R*_*0*_ for zero voltage and becomes saturated with increasing *V*_*app*_; the applied voltage that produces a relative brightness of 90% is termed the threshold voltage *V*_*th*_, and the applied voltage that produces a relative brightness of 10% is termed the saturation voltage *V*_*sa*_. The application of any voltage over *V*_*th*_ to the TN-LC cell causes the LC molecule to become vertically aligned. It can be seen (Fig. 4(a)) that in the voltage-off state (low voltage), the incident linearly polarised light passing through the front polariser changes its polarisation such that it is perpendicular to the front polariser following propagation through the TN-LC cell, and the light then reflects from the rear GBO polariser and passes again through the TN-LC cell and the front polariser. The reflectance is therefore high (*R*_*0*_ ~ 13–15%), representing the bright reflection state for *V*_*app*_ < *V*_*th*_ (0.75 V). Moreover, when the voltage applied to the TN-LC cell *V*_*app*_ > *V*_*th*_, the effective birefringence and retardation value decrease to zero, and the amount of reflected light decreases, resulting in light absorption by the bottom PSC. The results of the measurements were: *V*_*th*_ = 0.70 V, *V*_*sa*_ = 1.10 V, *R*_*0*_ = 15.1% (for G), and the absolute reflectance at *V*_*sa*_ was 0.9%. Therefore, the contrast ratio of the intensity of bright to dark (*I*_BRIGHT_/*I*_DARK_) increases, reaching a maximum value of *ca*. 18.1, 18.1, and 17.5, for R, G, and B light, respectively, *i.e.*, over a wide range of wavelengths of incident light. This contrast ratio clearly increases at high voltages, and the threshold voltage for the bright state is only about 0.75 V, which makes it suitable for a variety of applications, although the contrast ratio value at a given voltage differs slightly for each colour due to the variation in the phase retardation of the TN-LC cell. At the same time, we also assessed the power-generating abilities of the bottom PSC in terms of the short-circuit photocurrent from the Solar-LCD as a function of the voltage applied to the TN-LC cell in NW mode under B, G, and R light, as also shown in Fig. 4(a). Here it can clearly be seen that in the voltage-on state, the bottom PSC in the Solar-LCD generated an output photocurrent density *J*_*sc*_ of more than 0.40 mA/cm^2^ per unit of incident light power density (mW/cm^2^, green) for *V*_*app*_ above *V*_*sa*_ (1.10 V), because incident light passed through the GBO reflecting polariser, whereas *J*_*sc*_ decreased markedly to 0.05 mA/cm^2^ when *V*_*app*_ < 0.7 V. The measured response times are also shown in the inset of Fig. 4(a). The rising (field on) and falling (field off) times of the responses were found to be about 8.5 ms and 27.5 ms, respectively, meaning that the switching times are fast enough for video-rate applications.

In NB mode the reflectance and output photocurrent are inversely related to the applied voltage of the Solar-LCD cell, as shown in Fig. 4(b). For *V*_*app*_ = 0 V, the absolute reflectance *R*_*0*_ is low; the reflectance increases and becomes saturated (relative brightness of 100%) with increasing applied voltage; the applied voltage that produces a relative brightness of 10% is denoted *V*_*th*_, and the applied voltage that produces a relative brightness of 90% is denoted *V*_*sa*_. The results of the measurements were: *V*_*th*_ = 0.75 V, *V*_*sa*_ = 1.25 V, *R*_*0*_ = 0.7% (for G), and the absolute reflectance at *V*_*sa*_ was 12.6%. The contrast ratio had a maximum value of *ca*. 18.2, 19.4, 19.4 for B, G, and R light, respectively. We also showed that in the voltage-off state, the Solar-LCD generated a high output *J*_*sc*_ of 0.40 mA/cm^2^ per unit of incident light power density (mW/cm^2^, G) for *V*_*app*_ below *V*_*th*_ (0.75 V) because the incident light passes through the GBO reflecting polariser, resulting in light absorption by the bottom PSC, whereas *J*_*sc*_ decreased markedly to 0.04 mA/cm^2^ when the applied voltage exceeded *V*_*sa*_ (1.25 V). From these results, we confirm that the performance of the Solar-LCD with the GBO polariser and PSC was better in terms of brightness, contrast ratio, and power generating ability than has been reported in previous studies^4^,^5^,^6^.

**Figure 5:**
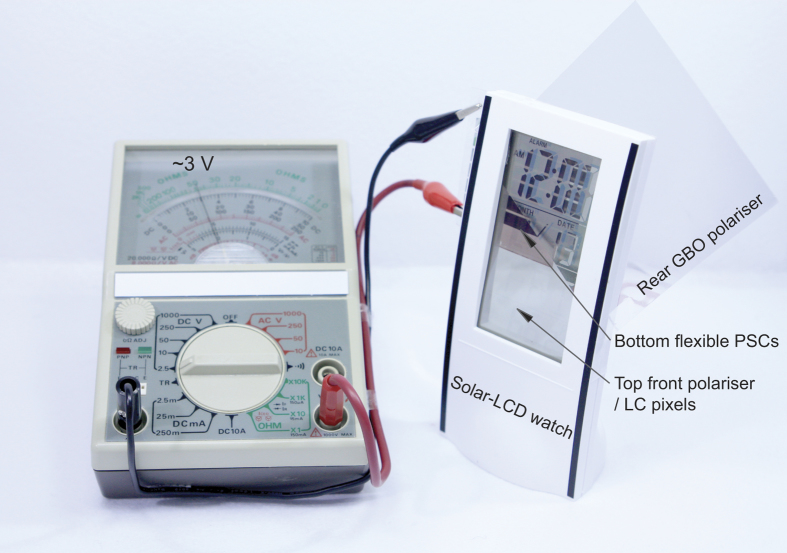
Photograph of a power-generating reflective-type bended Solar-LCD watch (right) displaying “12:00” in the NW mode under ambient indoor light, and a multimeter (left) for monitoring the power generated from the Solar-LCD. To illustrate the clear Solar-LCD structure, a front dichroic polariser and LC cell are shown partially overlapping on a flexible GBO reflective polariser and a PSC.

In order to demonstrate an application of our reflective-type Solar-LCD, we fabricated and tested a bended power-generating Solar-LCD watch, which we made by combining a flexible PCDTBT:PCBM_70_ PSC and a GBO reflecting polariser with a commercial TN-LCD watch containing a driving circuit. Figure 5 shows a bended reflective Solar-LCD watch in operation, displaying “12:00” in the NW mode, together with a multimeter for monitoring the power generated from the Solar-LCD. As shown in the photograph, the flexible light-absorbing PSC disappeared from the surface panel, eliminating problems caused by poor organisation in the design. The photograph also shows the clear visibilities of the reflective Solar-LCD watch under white illumination. Note that, for comparison, the final “0” is incomplete on the display due to the partial underlapping of the blackish bottom PSC beneath the LC display. Moreover, in addition to its clear visibility and high contrast, the Solar-LCD generated electricity from ambient coloured illumination, demonstrating its dual functionality as a power-generating full-colour display. The advantages of this system over previous Solar-LCDs, particularly for indoor use, lie in the clarity of the display and in its ability to generate electricity. These are the unique features of the reflective-type Solar-LCD proposed herein, which is capable of generating ~270 mW/m^2^ or more under typical indoor office conditions (ca. 400–500 lux, or ~5.8 W/m^2^), much higher than that (~100 mW/m^2^) of the LSC system reported^7^.

We now make two final comments on our Solar-LCD, the first of which relates to the improvement in its reflectance. In our device, we used a conventional dichroic polariser exhibiting an average transmittance of about 17% under visible light. However, had we instead used a high-contrast dichroic polariser, the reflectance could possibly have increased to more than 35%. Our second comment relates to the use of PSCs as the power-generating solar cell in our Solar-LCD. In place of the PSCs used in this study, it would be possible to use conventional inorganic semiconducting solar cells, e.g., amorphous or crystalline silicon solar cells. This could also yield highly efficient Solar-LCDs without any loss of their opto-electronic functionality as described above. However, some of the advantages of the PSCs could be sacrificed, including their mechanical flexibility, for example. Therefore, for flexible, bendable, or wearable Solar-LCDs, one of the candidates for the power-generating element is this PSC.

## Conclusions

We have described the opto-electrical and photovoltaic characteristics of a reflective-type Solar-LCD consisting of a TN-LC cell, a GBO reflective polariser, and an isotropic PCDTBT:PCBM_70_ PSC. The reflectivity of the Solar-LCD increased significantly to more than 13–15% under visible-light illumination, and exhibited a significantly improved contrast ratio of 17–19 with a high PCE of about 5%. These reflective Solar-LCDs could be used to make novel power-generating, flexible and reflective full-colour displays. Our results provide an encouraging starting point for the design of a variety of energy-harvesting opto-electrical display devices. Use of the Solar-LCD structure with the reflecting polariser reported here, together with highly efficient PV material systems reported elsewhere, could lead to the successful fabrication of highly effective and improved Solar-LCDs, rendering such devices acceptable in many energy-harvesting applications, including displays and/or optoelectronic devices.

## Additional Information

**How to cite this article**: Ho Huh, Y. and Park, B. Power generating reflective-type liquid crystal displays using a reflective polariser and a polymer solar cell. *Sci. Rep.* **5**, 11558; doi: 10.1038/srep11558 (2015).
